# 

**Published:** 1885-11

**Authors:** 


					﻿'hole Ni/mber, 311.
NOVEMBER, I885.
Vol. LI.. .Number 5
TZE3ZZE
CHICAGO MEDICAL JOURNAL AND EXAMINER
EDITORS:
S. J. JONES, M.D., LL.D.
W. W. JAGGARD. A M , M.D.
HAROLD N. MOYER, M.D
CHICAGO:
PUBLISHED MONTHLY BY THE CHICAGO MEDICAL PRESS ASSOCIATION
240 Wabash Avenue.
CLARK & LONGLEY PRINTERS, 163 AND 165 DEARBORN STREET, CHICAGO.
				

